# Direct numerical simulation of three-dimensional Kolmogorov flow for turbulence model development

**DOI:** 10.1038/s41597-026-06899-9

**Published:** 2026-02-26

**Authors:** Kinga Andrea Kovács, Miklós Balogh, Gergely Kristóf

**Affiliations:** Department of Fluid Mechanics, Faculty of Mechanical Engineering, Budapest University of Technology and Economics, Műegyetem rkp. 3., H-1111 Budapest, Hungary

**Keywords:** Mechanical engineering, Fluid dynamics

## Abstract

We present a curated dataset comprising three-dimensional direct numerical simulations of Kolmogorov flow across multiple distinct parameter regimes. These simulations cover a wide range of Reynolds numbers and excitation modes, capturing diverse flow behaviours. The dataset is designed to support research in turbulence modelling, particularly for machine learning-based model development. Each case includes raw velocity fields and a Python-based interpolation code is provided for the interpolation of the velocity fields onto a structured grid in a format compatible with ParaView, accompanied by comprehensive metadata for ease of use. This open-access dataset aims to facilitate advances in turbulence modelling, a continuing and vital challenge in fluid mechanics research.

## Background and Summary

Kolmogorov flow, a canonical configuration involving sinusoidal body forcing in a periodic domain, has long served as a simplified yet insightful model for studying hydrodynamic stability, non-linear dynamics, and turbulence^1^. Despite its idealized nature, it captures many essential features of real turbulent systems – such as anisotropy, intermittency, and complex transition dynamics – making it a valuable platform for both theoretical and computational fluid dynamics (CFD) research.

Introduced in the late 1950s^2^, the Kolmogorov flow has been extensively studied. Meshalkin and Sinai^3^ analysed it as a simple example of linear instability, while Platt *et al*.^4^ conducted numerical investigations of its chaotic regimes. Three-dimensional simulations were later performed by Shebalin and Woodruff^5^ and by Borue and Orszag^6^. The flow has also been experimentally realized utilizing magnetohydrodynamic forcing^7^, and later in soap films^8^. Batchaev and Ponomarev^9^ examined a laboratory model of the Kolmogorov flow and found that the specific type of secondary flow depends on the number of half periods in relation to the main flow.

Boffetta *et al*.^10^ investigated drag reduction in viscoelastic fluids using direct numerical simulations (DNS) of turbulent Kolmogorov flow, showing that drag reduction occurs above a critical Reynolds number. Musacchio and Boffetta^11^ further examined how turbulent drag depends on mean flow amplitude and conducted a scale-by-scale energy balance analysis to predict large-scale energy transport. The flow’s response to variations in Reynolds number, forcing scale, or additional physical effects – such as stratification or boundaries – has also been widely explored^12–15^.

More recently, DNS has enabled high-fidelity studies of turbulent regimes in Kolmogorov flow, contributing to understanding dissipation dynamics, energy spectra, and eddy-viscosity models^16–18^. Schaefer *et al*.^18^, for example, performed DNS at three Reynolds numbers and evaluated model equations for mean dissipation (*ε*) in the context of two-equation Reynolds-averaged Navier-Stokes (RANS) modelling, highlighting the potential of such data for improving turbulence closures. Menter and Egorov^19^ also utilized Kolmogorov flow for the parametrization of the KSKL turbulence model, which is the engine of the Scale-Adaptive-Simulation (SAS) method. Even though the name “Kolmogorov flow” was not explicitly stated by Menter and Egorov, it can be assumed that the periodic, sinusoidally-forced configuration they described is, in fact, Kolmogorov flow.

Despite its popularity as a model flow, publicly available, high-resolution datasets encompassing a wide parameter range remain scarce. The dataset presented in this work addresses this gap by providing a collection of three-dimensional DNS of Kolmogorov flow across a broad spectrum of Reynolds numbers and forcing configurations. Each case includes raw volumetric velocity fields and is supplemented with a Python-based interpolation utility for resampling the high-fidelity simulation data onto structured Cartesian grids. This utility ensures compatibility with common post-processing tools like ParaView and modern machine learning frameworks such as PyTorch or TensorFlow. By providing pre-processed, structured snapshots, this work eliminates the significant computational and technical overhead typically required to transform raw DNS results into a format suitable for algorithmic training, thereby making high-fidelity turbulence data accessible to a broader range of researchers who may not have access to large-scale high-performance computing resources.

This dataset is designed to support a wide range of applications, from basic studies of turbulence to the training and validation of reduced-order models and data-driven closure schemes. By offering a standardized, curated collection of Kolmogorov flow simulations, it contributes to ongoing efforts in reproducible research and accessible data sharing in CFD.

The practical utility of this dataset is underscored by the historical challenges associated with modelling Kolmogorov flow. Although it has been studied for decades, its potential as a rich source of information for turbulence model development has not been fully exploited. Standard eddy viscosity models, such as the *k*–*ε*^20^ and *k*–*ω* SST^21,22^ formulations, fail to capture even the qualitative features of Kolmogorov flow, typically producing diverging turbulent length scales and unphysically low velocities. One of the root causes of this might be that the Kolmogorov flow exhibits a strong dependence on the domain size, which directly constrains the turbulent length scale, a feature mirrored in many practical flows, such as atmospheric boundary layers.

Previous attempts to address such unphysical growth include the modified dissipation equations of Apsley and Castro^23^, which imposed a maximum mixing length. While other length-scale definitions based on velocity derivatives exist, they often suffer from numerical instability^18^. The Kolmogorov flow clearly demonstrates that relevant constraints can stem directly from the computational domain itself, necessitating scale-aware models that account for global geometric limits^24,25^.

Recently, the diverse parameter regimes in the present dataset were utilized to address these specific RANS modelling deficiencies. As reported by Kristóf *et al*.^26^, a new two-regime, geometry-informed (GI) eddy viscosity formulation was developed by identifying a sublinear geometric scale dependence. This model incorporates a power function dependent on the geometric filter size Δ to prevent the unbounded growth of the turbulent length scale. The proposed GI *k*–*ε* model, validated against these DNS data, outperformed traditional models by providing superior predictions of turbulent kinetic energy and mitigating turbulent inflation, thereby demonstrating the dataset’s value as a rigorous benchmark for turbulence model discovery.

## Methods

The DNS results were obtained using the NEK5000^27^ CFD solver, a highly scalable open-source spectral element method (SEM) code. In SEM, the solution is represented using high-order tensor-product polynomials with Gauss-Lobatto-Legendre (GLL) nodal bases within each element. SEM is characterized by minimal numerical dispersion and dissipation, making it suitable for both DNS and Large Eddy Simulation (LES).

The computational domain was a cube with side length 2Δ = 0.05 m. In each spatial direction, either 8 or 16 spectral elements were used, each with 16 GLL points for the velocity field, resulting in 128 or 256 points per direction. For dealiasing, 20 GLL points per element were employed. Periodic boundary conditions were imposed in all coordinate directions. The time step size varied throughout the simulations to maintain a maximum Courant number of 0.5. The simulations were initialized with a randomly perturbed velocity field. In all cases, low-pass (explicit) filtering was applied using two filter modes and a filter weight of 0.05. The fluid density was set to *ρ* = 1000 kg/m^3^, and the *μ* dynamic viscosity values for the different simulation cases are listed in Table 1.

**Table 1:** Simulation cases of the DNS reference database.

#	Flow type	${{\bf{Re}}}_{{\boldsymbol{\Delta }}}$	Mode	$\widehat{{\bf{f}}}\,{\boldsymbol{[}}{\bf{m}}/{{\bf{s}}}^{2}{\boldsymbol{]}}$	*μ* [*kg*/(*m* ⋅ *s*)]	Δ*t* [s]	Files	Original domain	Interpolated domain
1	DKF	997	1	0.2	0.001	0.25	20	128^3^	256^3^
2		499	1	0.2	0.002	0.25	20	128^3^	256^3^
3		997	1	0.2	0.001	0.25	25	256^3^	512^3^
4		1995	2	1.6	0.001	0.25	35	256^3^	512^3^
5		997	2	1.6	0.002	0.25	35	256^3^	512^3^
6		499	2	1.6	0.004	0.25	25	256^3^	512^3^
7	QSKF	1995	1	0.2	0.0005	0.05	50	256^3^	512^3^
8		499	1	0.2	0.002	0.05	100	128^3^	256^3^
9		125	1	0.2	0.008	0.05	300	128^3^	256^3^
10		997	1	0.2	0.001	0.25	100	128^3^	256^3^
11		499	1	0.2	0.002	0.25	100	128^3^	256^3^
12		249	1	0.2	0.004	0.25	70	128^3^	256^3^
13		125	1	0.2	0.008	0.25	100	128^3^	256^3^
14		499	1	0.8	0.004	0.125	100	128^3^	256^3^
15		1995	1	0.2	0.0005	0.25	50	256^3^	512^3^
16		997	1	0.2	0.001	0.25	50	256^3^	512^3^
17		1995	2	1.6	0.001	0.25	50	256^3^	512^3^
18		997	2	1.6	0.002	0.25	50	256^3^	512^3^
19		499	2	1.6	0.004	0.25	50	256^3^	512^3^

**Fig. 1:**
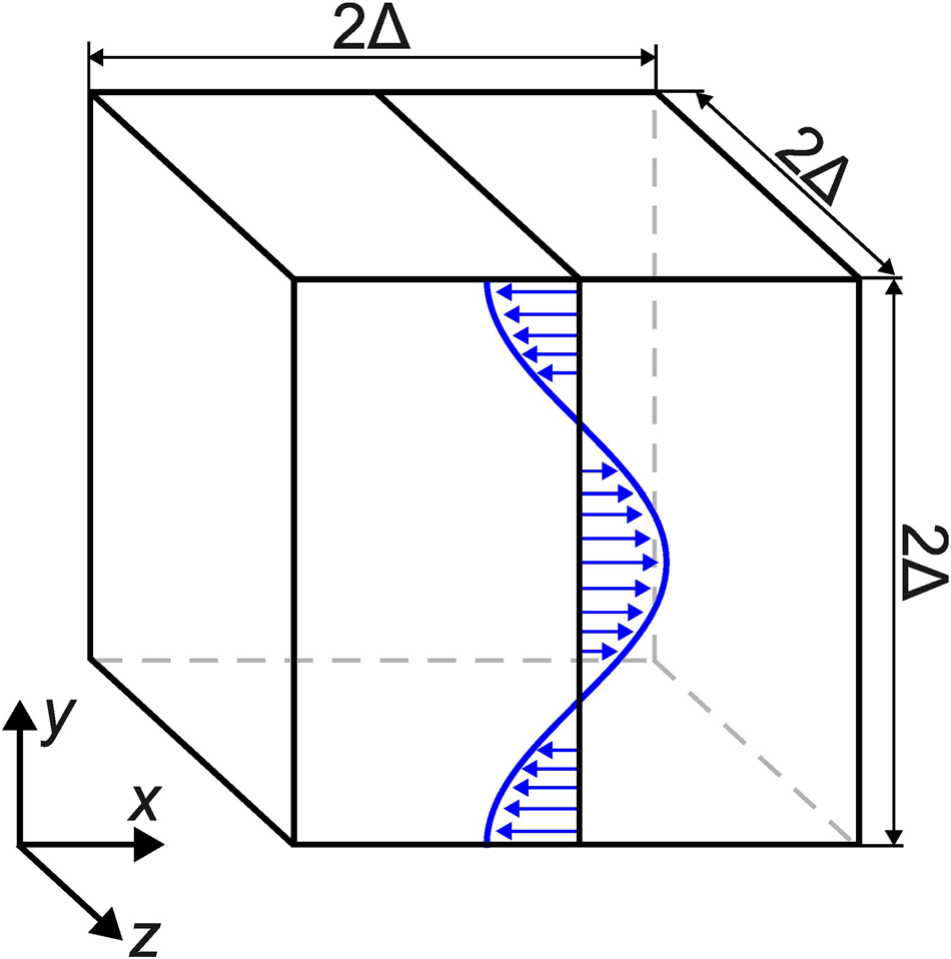
Spatial distribution of the driving force.

The *x*-direction driving force shown in Fig. 1 was implemented via a local acceleration subroutine (userf) as follows:

1 $${f}_{x}=-\widehat{f}\cos (\kappa y),$$

where $\widehat{f}$ is the force amplitude and *κ* is the excitation wave number. The Δ half-length of the domain defines the fundamental wave number of velocity modes in turbulent flow. For example, in Kolmogorov flow excited in the first mode, the wave number is *κ*_1_ = 2*π*/(2Δ) = *π*/Δ, and in Kolmogorov flow excited in the second mode *κ*_2_ = 4*π*/(2Δ) = 2*π*/Δ. For the evaluation of the DNS results the reference velocity was defined as the maximum value of the friction velocity:

2 $${U}_{\tau }=\sqrt{\frac{\widehat{f}}{\kappa }},$$

while the Reynolds number was defined in terms of the Δ half-length of the domain:

3 $${{\rm{Re}}}_{\Delta }=\frac{{U}_{\tau }\Delta }{\nu }.$$

where *ν* = *μ*/*ρ* is the kinematic viscosity.

**Fig. 2:**
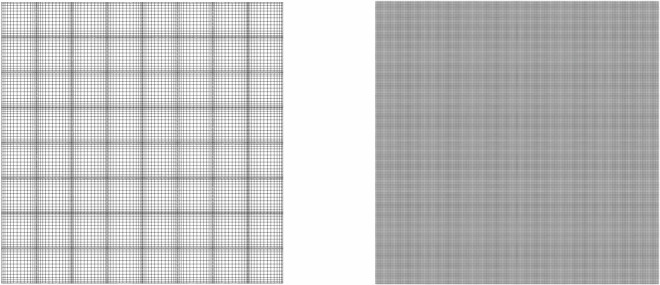
Non-equidistant original grid (left), and new equidistant grid (right).

For post-processing, the velocity fields were interpolated from the original grid (Fig. 2 left) onto a uniform mesh (Fig. 2 right) with 256^3^ or 512^3^ nodes using 15^th^-degree Lagrange polynomials. Velocity gradient components were then computed using a second-order central difference scheme. The interpolated fields were saved as .vtk files for visualization in ParaView. It is worth noting that the original NEK5000 output files can also be directly read by ParaView, provided a .nek5000 metadata file is available.

**Fig. 3:**
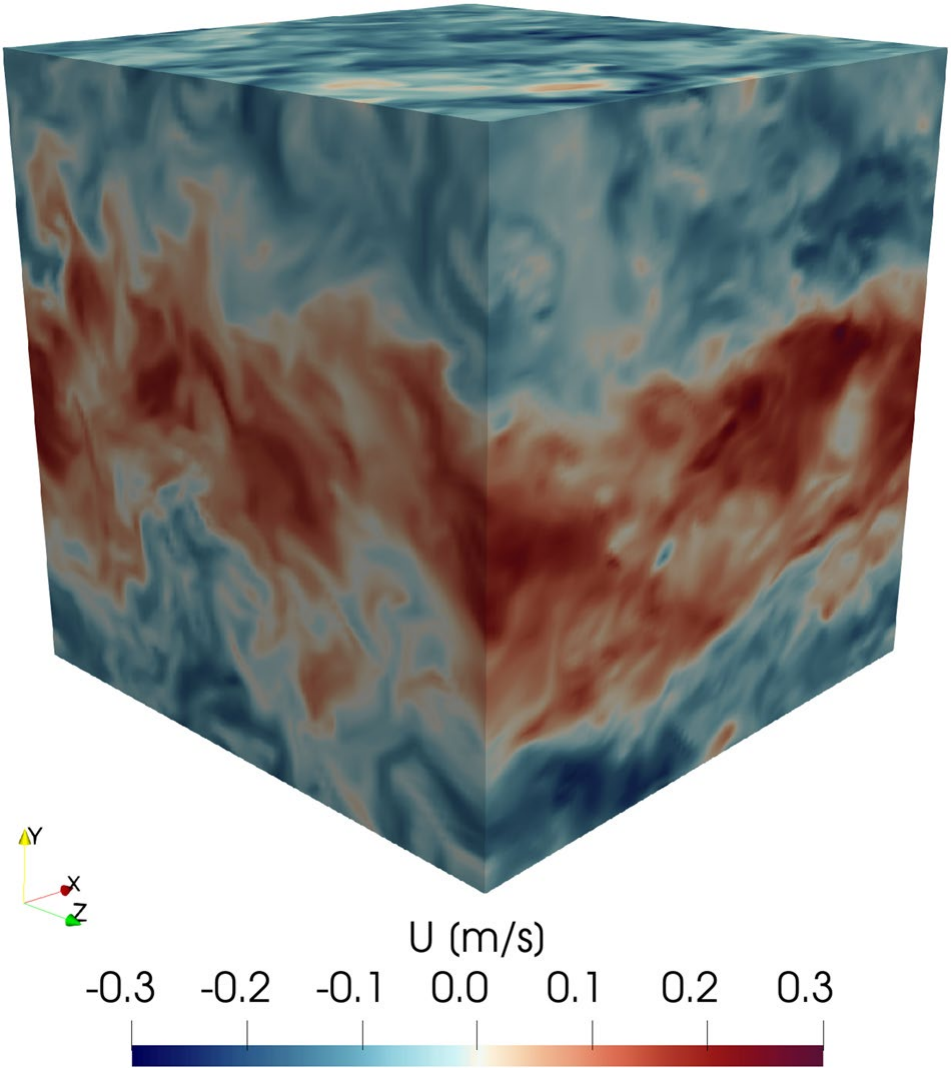
Instantaneous streamwise velocity distribution at t = 10.75 s for Case 10.

A detailed description of the different simulation cases is provided in Table 1, and in Fig. 3 the contour plot of the *x*-wise velocity can be seen for Case 10. In the table, DKF and QSKF refer to decaying and quasi-stationary Kolmogorov flow, respectively. In the DKF cases, the driving force was turned off after reaching a quasi-steady state, and ${{\rm{Re}}}_{\Delta }$ denotes the initial Reynolds number which decreases over time. In the table Δ*t* indicates the uniform time interval between saved files. The Files column lists the number of snapshot files available for each case. The Original domain column shows the resolution of the simulation domain, where 16 GLL points were used per element. The number of spectral elements was either 8 × 8 × 8 or 16 × 16 × 16, resulting in a total resolution of 128^3^ or 256^3^ points, respectively. The Interpolated domain refers to the resolution after interpolating the results onto an equidistant grid.

During post-processing, bulk flow variables are defined as layer-averaged quantities obtained by averaging instantaneous fields over the *x*-*z* planes. Consequently, all characteristic turbulence quantities – bulk velocity, strain rate, turbulent kinetic energy, production, and dissipation – become functions of *y* and *t*. These averaged quantities are directly comparable with one-dimensional unsteady Reynolds-averaged flow models; however, the finite size of the computational domain limits the statistical convergence of turbulent quantities obtained from DNS. The bulk flow quantities exhibit temporal fluctuations that must be consistently represented by the corresponding unsteady RANS model. After computing the RANS model predictions and prior to comparison with DNS reference data, a secondary averaging procedure is recommended.

Secondary averaging can be performed either in the wall-normal (*y*) direction – denoted by the subscript *t* – or over time, yielding spatial profiles – denoted by the subscript *y*. This projection method reduces statistical noise associated with finite sampling and enables both spatially resolved (*y*-dependent) and temporally resolved (*t*-dependent) analyses. When no secondary averaging subscript is specified, only the initial layer averaging is implied. In contrast, when averaging is applied in all directions – layer averaging followed by averaging in both *y* and *t* – the resulting fully averaged quantity is denoted by an overbar.

The primary layer-averaging strategy adopted here represents one suitable choice for data reduction. Alternative primary filtering approaches, such as clustering-based averaging over cubic subdomains, are also viable but are not considered further in order to keep the scope of the present data descriptor focused on providing a consistent reference processing methodology.

## Data Records

The three-dimensional DNS data for Kolmogorov flow were made publicly accessible^28^. To facilitate data acquisition, the folder structure is shown below. The .zip folders were uploaded separately for the different simulation cases. The suggested structuring of the data for the interpolation is detailed in the *Usage Notes* section.

The shear_3d.nek5000 metadata file should be used to read the shear_3d0.fxxxxx NEK5000 files in ParaView. These files contain the *x*-, *y*-, and *z*-wise components of velocity, the velocity magnitude, the pressure, and the real flow-time data on the original, non-equidistant grid. To obtain equidistant grid data, the Resample to Image filter in ParaView can be applied. However, this approach does not provide sufficient accuracy for tasks such as turbulence model development. Therefore, to ensure highly accurate equidistant grid data, the interpolation mentioned above was performed in Python. The resulting velocity fields were saved in .vtk format. These files, due to their large sizes, are not included in the dataset presented herein, however, the interpolation code can be accessed at a public Git repository at https://github.com/KovacsKA00/Nek5000-interpolation.git, thus enabling users to generate their own .vtk files. This is detailed in the *Usage Notes* and *Code Availability* sections.

## Technical Validation

**Fig. 4:**
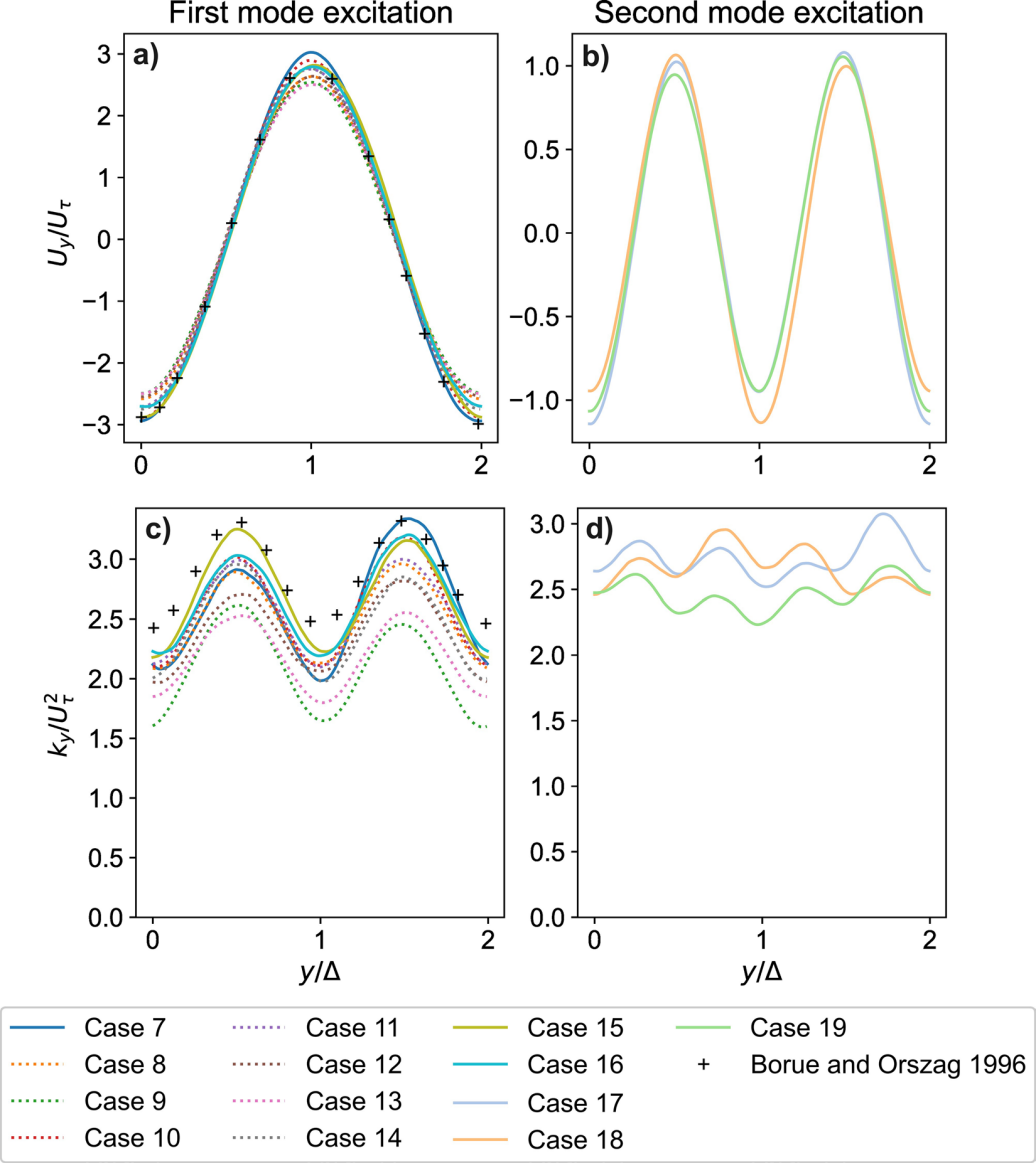
Comparison of time-averaged velocity - (**a**) and (**b**) - and turbulent kinetic energy - (**c**) and (**d**) - profiles with the results of Borue and Orszag^6^.

In the numerical flow model of Borue and Orszag^6^, hyperviscosity was employed, enabling the computation of Reynolds number-independent solutions on grids with a resolution comparable to that used in the present study. The maximum value of the effective Reynolds number, computed from the Taylor microscale, was approximately ${{\rm{Re}}}_{\lambda }\approx 1000$. For the quasi-stationary Kolmogorov flow, the time-averaged and normalized mean velocity (*U*_*y*_) and turbulent kinetic energy (*k*_*y*_) profiles are compared with the Reynolds number-independent reference data of Borue and Orszag in Fig. 4.

It should be noted that in the work of Borue and Orszag^6^ the reference velocity *U*_*τ*_ differs from the definition in Eq. (3) by an arbitrary scaling factor of 2.5. Therefore, the dimensionless velocity and *k* profiles taken from the work of Borue and Orszag^6^ were multiplied by factors of 2.5 and 6.25, respectively, to ensure consistency in the comparisons. Figure 4(a),(b) show that the mean velocity profile exhibits only a weak dependence on the Reynolds number within the investigated range. As expected, our results converge towards the reference profiles of Borue and Orszag^6^ as the Reynolds number increases.

To assess the spectral accuracy of our numerical model, we compared our results with the universal equilibrium *k* spectrum proposed by Pope^29^. The instantaneous *k* spectra were obtained from the velocity field by computing the power spectra using a fast Fourier transform, integrating the spectral energy over spherical shells of constant thickness in wave number space, and subtracting the energy spectrum of the bulk-flow velocity profiles derived from *x*-*z* plane-averaging.

**Fig. 5:**
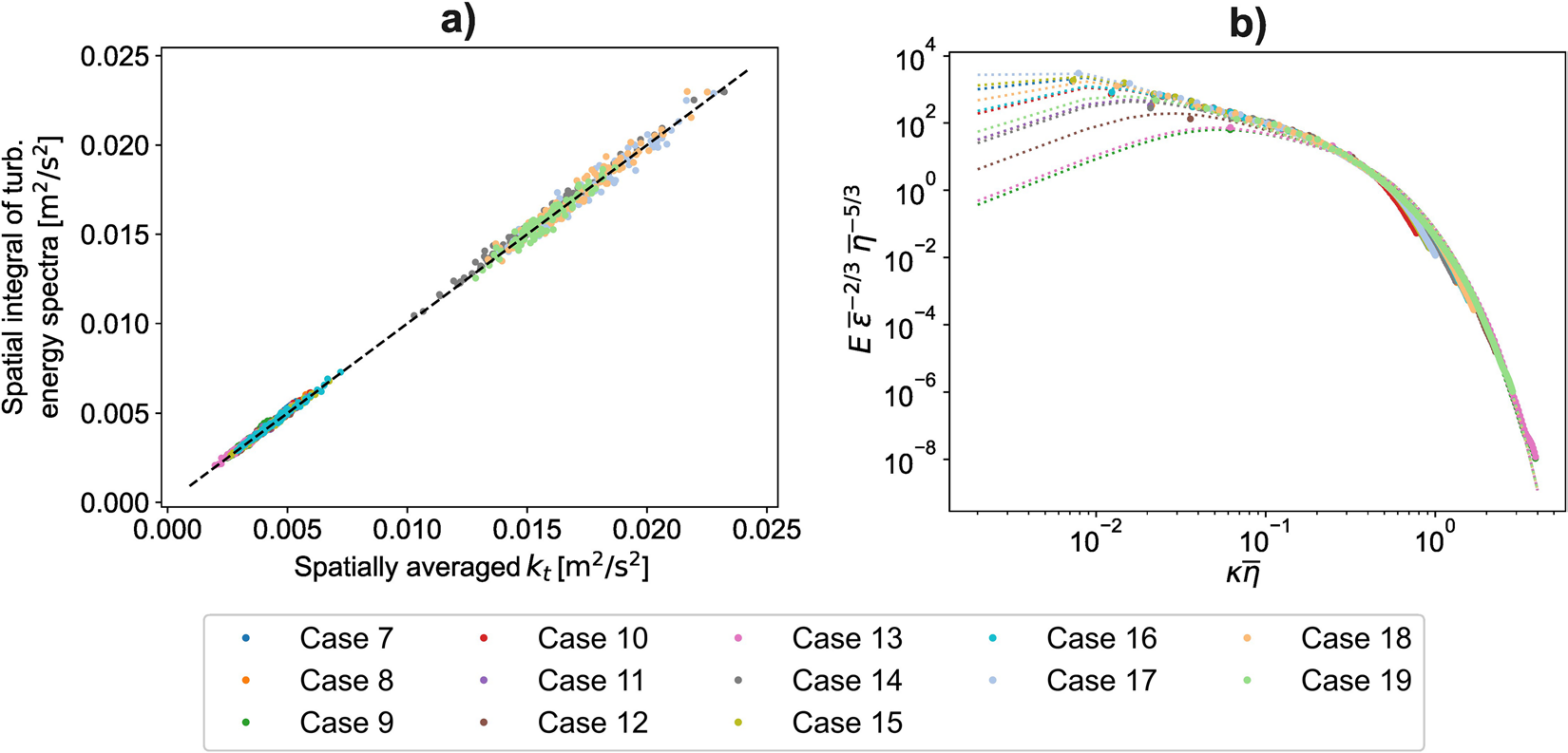
Validation by examining the energy spectra: (**a**) energy check - spatially integrated spectra match the turbulent kinetic energy from the velocity field (1 : 1 line); (**b**) normalized radial spectra - 3D spectra integrated in spherical shells and scaled by Kolmogorov units collapse at high *kη*, showing universal small-scale behaviour.

The total energy of the instantaneous spectra was verified against *k*_*t*_ directly computed from the velocity field, with the results shown in Fig. 5(a). The time-averaged energy spectra presented in Fig. 5(b) were non-dimensionalised using the Kolmogorov length scale, $\overline{\eta }$, to examine possible distortions in the high-wave-number range. For the original grid with 128^3^ nodes, 64 spectral components were displayed, while for the 256^3^ grid, 128 spectral components were shown. Our results show good agreement with the equilibrium spectrum across the entire wave number range.

For the coarser grid, however, parts of the spectrum fall below the reference spectrum near the Kolmogorov scale $\left(\overline{k}\overline{\eta }\approx 1\right)$, indicating an under-resolved numerical simulation. Due to the small energy content of the fine-scale structures, the limited resolution had only a minor impact on the *k* and turbulent production values. Nevertheless, a noticeable deficit in turbulent dissipation was observed for the under-resolved cases. To avoid systematic modelling errors, this deficit can be compensated by examining the energy balance, specifically the equality:

4 $$\overline{\varepsilon }=\overline{P-\frac{{\rm{d}}k}{{\rm{d}}t}},$$

where *ε* and *P* are the dissipation and production of the turbulent kinetic energy *k*, respectively.

Examining the energy balance is essential, as it verifies the consistency of the resolved turbulent quantities. While the numerical resolution of our Kolmogorov flow simulations was generally sufficient to fully resolve the turbulent kinetic energy and its production, in some cases a dissipation deficit was revealed. In quasi-stationary Kolmogorov flow, the distribution of turbulent scales is nearly homogeneous, thus enabling a correction of the dissipation rate based on the averages of the well-resolved quantities.

The corrected dissipation *ε* is estimated from the resolved dissipation *ε*_*r*_ using a dissipation correction factor (*DCF*):

5 $$\varepsilon =\frac{{\varepsilon }_{r}}{DCF},$$

where *ε*_*r*_ is extracted directly from the velocity field according to its definition, and the factor *DCF* is computed from averaged quantities:

6 $$DCF=\frac{\overline{{\varepsilon }_{r}}}{\overline{P-\frac{{\rm{d}}k}{{\rm{d}}t}}}.$$

Table 2 shows the *DCF* values for the quasi-stationary simulations alongside the Kolmogorov length scale $\overline{\eta }$ and the grid resolution Δ*x*. For cases with lower ${{\rm{Re}}}_{\Delta }$, the $\Delta x/\overline{\eta }$ values indicate a well-resolved DNS, and the corresponding *DCF* values are exactly unity. This confirms that these cases are fully resolved down to the Kolmogorov scale, capturing the entire dissipation spectrum without numerical truncation. For higher Reynolds number cases where the grid resolution becomes relatively coarser, the *DCF* is slightly smaller than unity to compensate for energy truncation at the grid level. Applying this correction ensures consistency across the dataset and significantly reduces systematic errors in the reconstruction of the dissipation rate in under-resolved cases.

**Table 2:** *DCF* values and resolution ratios for the different simulation cases.

#	Flow type	${\mathrm{Re}}_{\varDelta }$	*DCF*	Δ*x*[m]	$\overline{\eta }[m]$	$\varDelta x/\overline{{\boldsymbol{\eta }}}$
1	DKF	997	—	—	—	—
2		499				
3		997				
4		1995				
5		997				
6		499				
7	QSKF	1995	0.86	0.00020	0.00006	3.42
8		499	0.95	0.00039	0.00017	2.34
9		125	1.00	0.00039	0.00049	0.80
10		997	0.82	0.00039	0.00010	4.03
11		499	0.95	0.00039	0.00017	2.37
12		249	0.99	0.00039	0.00028	1.38
13		125	1.00	0.00039	0.00049	0.80
14		499	0.94	0.00039	0.00017	2.37
15		1995	0.86	0.00020	0.00006	3.39
16		997	0.95	0.00020	0.00010	2.00
17		1995	0.87	0.00020	0.00006	3.14
18		997	0.96	0.00020	0.00010	1.87
19		499	0.98	0.00020	0.00018	1.10

**Fig. 6:**
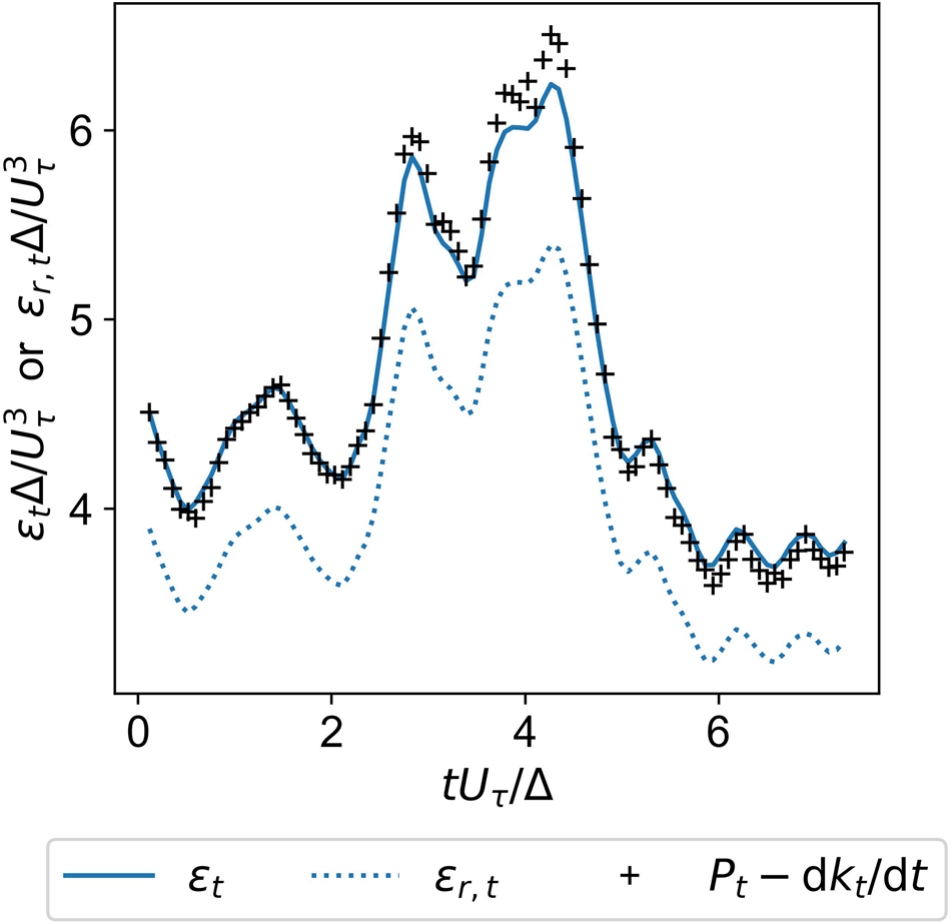
Resolved (*ε*_*r*,*t*_) and corrected (*ε*_*t*_) dissipation as a function of dimensionless time compared with the reference data (+ symbols) obtained from the energy balance for Case 7.

Figure 6 illustrates the effect of the dissipation correction for the case with one of the lowest *DCF* values. In Fig. 6 the *t* subscript denotes that both layer averaging and secondary averaging along the *y* coordinate – yielding a temporal profile – were performed.

In contrast with the turbulent kinetic energy dissipation, turbulent kinetic energy production is well resolved on the mesh. The accuracy of statistics is still limited by the finite length of the flow history. As demonstrated in Figure 9 of Kristóf *et al*.^26^, the convergence of the dimensionless turbulent production reaches a coefficient of variation of approximately ±2.5%. This level of residual variability is small relative to the qualitative shifts in flow behaviour across parameter regimes.

## Usage Notes

After downloading the different .zip folders for the separate simulation cases, the cases should be unwrapped under a common folder for the easiest use of the interpolation file. The proposed folder structure is the following:

The Python script for interpolation is provided in a public Git repository. The script interpolation.py converts .nek5000 output files into .vtk format. After setting the folder names, spatial dimensions, and some additional parameters, the script can process all cases automatically, saving the generated .vtk files into their corresponding data directories.

## Data Availability

The three-dimensional DNS dataset for Kolmogorov flow^28^ were made publicly accessible on Zenodo at https://zenodo.org/records/16733422 under the Creative Commons Attribution 4.0 International License (CC BY 4.0) (https://creativecommons.org/licenses/by/4.0/).

## Code availability

The custom Python script used in this study is available under the GNU General Public License v3.0. The Python code for interpolating simulation output is publicly accessible in a Git repository at https://github.com/KovacsKA00/Nek5000-interpolation.git. The repository includes:

•interpolation.py for interpolating .nek5000 output onto a uniform grid and exporting the data in .vtk format,

• a README file with instructions for setup and usage.

For the current study ParaView 5.13.3 and Python 3.10.11 were used.
